# Effectiveness of Workplace Interventions in Return-to-Work for Musculoskeletal, Pain-Related and Mental Health Conditions: An Update of the Evidence and Messages for Practitioners

**DOI:** 10.1007/s10926-016-9690-x

**Published:** 2017-02-21

**Authors:** K. L. Cullen, E. Irvin, A. Collie, F. Clay, U. Gensby, P. A. Jennings, S. Hogg-Johnson, V. Kristman, M. Laberge, D. McKenzie, S. Newnam, A. Palagyi, R. Ruseckaite, D. M. Sheppard, S. Shourie, I. Steenstra, D. Van Eerd, B. C. Amick

**Affiliations:** 1 Institute for Work & Health, 481 University Ave, Toronto, ON M5G 2E9 Canada; 2 Institute for Safety Compensation and Recovery Research, Monash University, Melbourne, VIC Australia; 3 School of Public Health and Preventive Medicine, Monash University, Melbourne, VIC Australia; 4 National Centre for Occupational Rehabilitation, Rauland, Norway; 5 Team WorkingLife ApS, Copenhagen, Denmark; 6 Department of Community Emergency Health and Paramedic Practice, Monash University, Melbourne, VIC Australia; 7 Lakehead University, Thunder Bay, ON Canada; 8 University of Montreal and CHU Ste-Justine Research Centre, Montreal, QC Canada; 9 Accident Research Centre, Monash University, Melbourne, VIC Australia; 10 Ted Rogers School of Management, Ryerson University, Toronto, ON Canada; 11 School of Public Health and Health Systems, University of Waterloo, Waterloo, ON Canada; 12 Robert Stempel College of Public Health and Social Work, Florida International University, Miami, FL USA

**Keywords:** Return to work, Workplace, Program effectiveness, Musculoskeletal pain, Mental health, Systematic review

## Abstract

*Purpose* The objective of this systematic review was to synthesize evidence on the effectiveness of workplace-based return-to-work (RTW) interventions and work disability management (DM) interventions that assist workers with musculoskeletal (MSK) and pain-related conditions and mental health (MH) conditions with RTW. *Methods* We followed a systematic review process developed by the Institute for Work & Health and an adapted best evidence synthesis that ranked evidence as strong, moderate, limited, or insufficient. *Results* Seven electronic databases were searched from January 1990 until April 2015, yielding 8898 non-duplicate references. Evidence from 36 medium and high quality studies were synthesized on 12 different intervention categories across three broad domains: health-focused, service coordination, and work modification interventions. There was strong evidence that duration away from work from both MSK or pain-related conditions and MH conditions were significantly reduced by multi-domain interventions encompassing at least two of the three domains. There was moderate evidence that these multi-domain interventions had a positive impact on cost outcomes. There was strong evidence that cognitive behavioural therapy interventions that do not also include workplace modifications or service coordination components are not effective in helping workers with MH conditions in RTW. Evidence for the effectiveness of other single-domain interventions was mixed, with some studies reporting positive effects and others reporting no effects on lost time and work functioning. *Conclusions* While there is substantial research literature focused on RTW, there are only a small number of quality workplace-based RTW intervention studies that involve workers with MSK or pain-related conditions and MH conditions. We recommend implementing multi-domain interventions (i.e. with healthcare provision, service coordination, and work accommodation components) to help reduce lost time for MSK or pain-related conditions and MH conditions. Practitioners should also consider implementing these programs to help improve work functioning and reduce costs associated with work disability.

**Electronic supplementary material:**

The online version of this article (doi:10.1007/s10926-016-9690-x) contains supplementary material, which is available to authorized users.

## Introduction

Despite overall work injury rates declining in most high-income countries [1, 2], equivalent improvements in return-to-work (RTW) rates (i.e. percentage returning to work within certain disability duration windows) have not been observed. In Australia and New Zealand, the latest data indicate RTW rates have remained static for 15 years [3]. Canadian-wide statistics comparing the percentage of wage loss claims at specific durations (e.g., 30 or 180 days after injury) indicate that disability duration has remained constant or increased between 2000 and 2008 [4]. Societal changes are making improvements in RTW more difficult to achieve. The ageing workforce poses particular challenges given findings that older workers take longer to RTW than younger workers and are more likely to ‘relapse’ into a period away from work following an initial return to work [5]. Similarly, there is a growing trend in precarious employment relationships (e.g., workers with short-term contract arrangements). Workers with precarious job arrangements also take longer to RTW than those with secure employment relationships [6].

There is now a substantial research literature on RTW interventions delivered in the workplace. This diverse literature contains relatively few high quality intervention studies. One systematic review of workplace based interventions published in 2004, for workers with musculoskeletal (MSK)- and pain-related conditions, identified ten good quality intervention studies after completing a search that retrieved 35 relevant studies [7]. The review found strong evidence that time away from work (work disability duration) is reduced by work accommodation offers and contact between healthcare providers and the workplace, and moderate evidence that other disability management interventions were effective. There was limited or mixed evidence of the impact of these interventions on health related quality of life.

The complex nature of interventions in this field poses a direct challenge for researchers. Conducting high-quality work disability research, and in particular, evaluating return-to-work interventions which have many socio-legal aspects and often requires the endorsement and cooperation of stakeholders with competing interests (e.g., employers, insurers, labour unions, provider networks, compensation authorities, etc) is difficult [8]. Still, in the decade since the review’s publication, and other studies by the same research team [9], there has been steady growth in the volume and scope of RTW intervention studies published. RTW or work disability research has emerged as a stand-alone field of endeavour encompassing multiple disciplines, with a rapidly growing evidence base [10].

This is true for both MSK and pain-related conditions; and more recently mental health (MH) conditions. The growth in literature focused on interventions to manage depression in the workplace has grown substantially over the last 5 years. In 2010, several authors from this research team published a systematic review [11] on interventions to manage depression in the workplace, finding 12 high quality studies. Recently, this team has sought to update findings on this question and have found the body of relevant literature to have more than doubled in the last 5 years (unpublished data).

Consistent with the best practice of updating systematic reviews as new evidence emerges [12], we sought to update and extend the previous review of workplace based RTW interventions that was limited to MSK and pain-related conditions. The primary objective of this review was to synthesize evidence on the effectiveness of workplace-based RTW interventions that assist workers with MSK, mental health (MH), and pain-related conditions to return to work after a period of work absence. The focus of this update was expanded to include MH conditions, based largely on input from our occupational health and safety (OHS) stakeholders given that the burden associated with managing the effects of mental health conditions in the workplace is extensive [13–16]. A particular strength of the Institute for Work & Health (IWH) systematic review program is the unique process of stakeholder engagement adopted throughout the review process [17]. Our stakeholders provide guidance to ensure the review question is relevant, the search terms are comprehensive and the targeted literature identified is up-to-date. But more importantly, stakeholders helped us examine the findings from this review to determine the best wording for our key messages to facilitate uptake and dissemination of these evidence-based approaches for OHS practitioners and other workplace parties This paper focuses on the evidence on RTW outcomes. A future paper will address the evidence from this review on recovery outcomes.

## Methods

The systematic review followed the six review steps developed by the Institute for Work & Health (IWH) for OHS prevention reviews [18]: (1) question development, (2) literature search, (3) relevance screen, (4) quality appraisal, (5) data extraction, and (6) evidence synthesis. The review team consisted of 17 researchers from Australia, Canada, Europe and the United States. Reviewers were identified based on their expertise in conducting epidemiologic or intervention studies related to work-related conditions, their experience in conducting systematic reviews or their clinical expertise. Review team members had backgrounds in epidemiology, ergonomics, kinesiology, physical therapy, psychology, social sciences, and information science. All 17 team members participated in all review steps.

The IWH Systematic Review program follows an integrated stakeholder engagement model during reviews [17]. Stakeholder meetings were held on multiple occasions through the review process in Toronto, Canada and Melbourne, Australia. Stakeholders were selected from injured worker advocacy groups, unions, workplaces, and health and safety associations and provided valuable input on search terms, inclusion/exclusion criteria, operational definitions, terminology, other search considerations, how findings of the review might be used, potential audiences, how the finalized review could be presented, how the review findings could be disseminated, and stakeholder information and communication needs throughout the review process.

### Question Development

The review team and stakeholders participated in a meeting to discuss the review update research question, and proposed search terms. The review question and search terms from the original review were used as a starting point and were updated through this process of question development. The inclusion of MH conditions to the final research question was an addition driven largely in response to stakeholder feedback through this process.

### Literature Search

Search terms were developed iteratively by the research team in consultation with a librarian, content area experts and stakeholders. Search terms were identified for three broad areas; population terms for workers and for injury/conditions, intervention terms, and outcome terms. Both database-specific controlled vocabulary terms and keywords were included. The terms within each category were combined using a Boolean OR operator and then terms across the three main categories were combined using a Boolean AND operator. The complete list of terms used in our search is reported in Supplementary Table 1.

The following electronic databases were searched; Medline, EMBASE, CINAHL, PsycINFO, Sociological Abstracts, Applied Social Sciences Index and Abstracts (ASSIA), and ABI Inform (American Business Index) from 1990 to April 2015. Research prior to 1990 was considered informative from a historical perspective but less relevant to current personal injury-illness compensation and other health care system and therefore excluded from this review. As the controlled vocabulary and the ability to handle complicated multi-term searches differ across the databases searched, search terms were customized for each database as required. All peer-reviewed literature was included, including non-English citations.

In addition to the database searches, the review team identified, from their own holdings and via contact with international content area experts, a list of studies that were in press or otherwise forthcoming in the published peer review literature.

References were loaded into commercially available review software (DistillerSR^®^) [19], which was also used for all remaining review steps. DistillerSR^®^ is an online application designed specifically for the screening, quality appraisal and data extraction phases of a systematic review.

### Relevance Screen

The review team devised five screening criteria to exclude articles not relevant to our review question: (a) commentary/editorial, (b) study was not about RTW or disability management/support, (c) non-intervention studies or interventions that did not occur as part of a system, program, policy or work practice change, (d) interventions that were not workplace-based, and (e) study population included greater than 50% of any of the following excluded conditions: severe traumatic brain injury, spinal cord injury, severe lower limb traumatic injuries including amputations; MSK disorders secondary to cancer, cancer-related pain or osteoporosis; and severe mental disorders (i.e. bipolar disorder, chronic severe depression or schizophrenia).

First, titles and abstracts of references were screened by a single reviewer. To limit the possibility of bias, a quality control (QC) step was implemented. A QC reviewer independently assessed a randomly chosen set of 329 titles and abstracts (approximately 5% of references from the search). Comparing the QC reviewer responses directly to review team responses, 27 conflicts (8%) (i.e. where the QC reviewer disagreed with the assessment of the original reviewer) were found. However, only four (1.2%) were conflicts in which the review team excluded references and the QC reviewer included them. The small (1.2%) number of consequential discrepancies suggests that reviewers had a similar understanding and application of the screening criteria.

Second, the full text of articles that advanced through the title and abstract screening process were screened using the same criteria, with two reviewers independently reviewing and coming to consensus. When consensus could not be reached, a third reviewer was consulted.

### Quality Appraisal

Relevant articles were appraised for methodological quality. The team grouped multiple articles associated with a single study, designating one article as the primary article. Study quality was assessed using 25 methodological criteria within the following broad headings: Design and Objectives, Level of Recruitment, Intervention Characteristics, Intervention Intensity, Outcomes, and Analysis (see Supplementary Table 2).

Methodological quality scores for each study were based on a weighted sum score of the quality criteria (with a maximum score of 96). The weighting values assigned to the 25 criteria ranged from ‘‘somewhat important’’ (1) to ‘‘very important’’ (3). Each study received a quality ranking score by dividing the weighted score by 96 and then multiplying by 100. The quality ranking was used to group studies into three categories: high (>85%), medium (50–85%) and low (<50%) quality [20].

Each study was independently assessed by two reviewers, who were required to reach consensus. Where consensus could not be achieved, a third reviewer was consulted. Team members did not review articles they had consulted on, authored or co-authored.

The quality appraisal represents an assessment on: internal validity, external validity, and statistical validity [21]. A higher quality score increases the team’s confidence that an effect was an intervention consequence rather than the effect(s) of other workplace or external environment factors. Therefore, data extraction and evidence synthesis were only completed on high and medium quality studies.

### Data Extraction

Standardized forms based upon previous reviews were used for data extraction [7, 11]. Extracted data were used to create summary tables sorted by intervention category and used for evidence synthesis. Data were extracted independently by pairs of reviewers. As in the relevance and quality appraisal stages, reviewer pairs were rotated to reduce bias. Team members did not review articles they consulted on, authored or coauthored. Any conflicts between reviewers were resolved by discussion. Stakeholders were consulted to determine relevant workplace-based RTW intervention categories.

### Evidence Synthesis

The evidence synthesis approach [18, 22] considers the quality, quantity and consistency in the body of evidence (see Table 1). First, the intervention categories created in the data summary tables were examined by the entire team. Once consensus was reached on the categories, the team moved to summarizing the evidence for each intervention category. Due to the heterogeneity of outcome measures, study designs and reported data, we chose not to calculate a pooled effect estimate. To determine individual study intervention effects, the following rules were applied: an intervention with a positive and no negative results was classified as a positive effect, an intervention with both positive and no effects was also classified as a positive effect intervention, an intervention with only no effects was classified as no effect, an intervention with any negative effect was classified as negative effect. Intervention effects were combined with the quality rating and number of studies to determine the level of evidence for each intervention category.

**Table 1:** Best evidence synthesis algorithm/algorithm for messages

Level of evidence	Minimum quality^a^	Minimum quantity	Consistency	Strength of message
Strong	High (H)	3	3H agree; if 3+ studies, ≥3/4 of the M and H agree	Recommendations
Moderate	Medium (M)	2H or 2H and 1M	2H agree or 2M and 1H agree; if 3+, ≥2/3 of the M and H agree	Practice considerations
Limited		1H or 2M or 1M and 1H	2 (M and/or H) agree; if 2+, >1/2 of the M and H agree	Not enough evidence to make recommendations or practice considerations
Mixed		2	Findings are contradictory	
Insufficient	Medium quality studies that do not meet the above criteria			

^a^High = >85% in quality assessment; medium = 50–85% in quality assessment

To generate practical messages, an algorithm developed by IWH along with OHS stakeholders was followed [23]. A strong level of evidence leads to “recommendations”. A moderate level of evidence leads to “practice considerations”. For all evidence levels below moderate, the consistent message is: “Not enough evidence from the scientific literature to guide current policies/practices”. This does not mean that the interventions with limited, mixed, or insufficient evidence may not be effective; only that there is not enough scientific evidence to draw conclusions.

## Results

### Literature Search

**Fig. 1:**
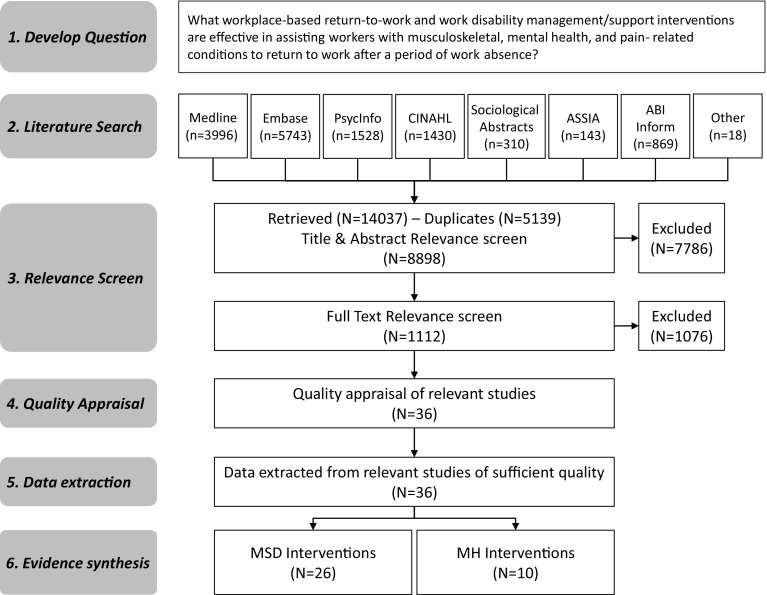
Flowchart of study identification, selection and synthesis

The search (covering 1990 to April 2015) identified 8880 references once results from the different electronic databases were combined and duplicates removed (Fig. 1). Eighteen additional papers not captured by the search were identified by the research team resulting in a total of 8898 references (Fig. 1).

### Relevance Screen

Overall, 7786 references and 1076 full articles were excluded for not meeting relevance criteria (reference list is available from corresponding author upon request). There were 36 unique studies (described in 65 articles) identified as relevant workplace-based interventions (Fig. 1), 26 of these examined interventions for MSK and pain-related conditions and 10 were focused on MH conditions.

### Quality Appraisal

Eighteen studies were classified as high quality (>85% of criteria met) [24–60] and 18 studies were medium quality (50–85% of criteria met) [61–92]. No studies were rated as low quality (<50% of criteria met) (Supplementary Table 2). The quality criteria that differentiated medium and high quality studies were non-randomisation and lack of allocation concealment (N = 16), substantial loss to follow up (N = 15), uneven attrition between groups (N = 22), lack of evidence of intervention compliance (N = 21), failure to blind participants and/or personnel (N = 27) and use of non-optimal statistical analyses (N = 13). Fifteen studies also failed to state clearly the primary study hypothesis (N = 15).

### Data Extraction

#### Study Characteristics

The study designs included randomized controlled trials (n = 19), non-randomized controlled trials (n = 7) and cohort studies with either concurrent (n = 4), historical (n = 4) or both concurrent and historical comparison groups (n = 2).

The studies came from the Netherlands (n = 11), USA (n = 6), Sweden (n = 6), Canada (n = 4), Finland (n = 2), Germany (n = 2), Australia (n = 1), Denmark (n = 1), Hong Kong (n = 1), UK (n = 1) and one multi-jurisdictional study which included participants in Denmark, Germany, Israel, the Netherlands, Sweden and USA.

The sectors included public administration (n = 2), professional, scientific or technical services (n = 3), mining (n = 1), construction (n = 2), agriculture (n = 2), manufacturing (n = 10), transportation (n = 3), health care and social assistance (n = 17), educational services (n = 3), hospitality and other services (n = 5), other (n = 5), and unknown (n = 13). Some studies included populations from multiple sectors.

The length of follow-up in these studies ranged from 4 weeks to 10 years, with the majority (N = 17) having a 12-month follow-up. Other lengths of follow-up observed in these studies included 4 weeks (N = 1), 8 weeks (N = 1), 6 months (N = 2), 14 months (N = 1), 18 months (N = 3), 2 years (N = 5), 3 years (N = 3), 6 years (N = 2), and 10 years (N = 1).

Study characteristics can be found in Table 2.

**Table 2:** Characteristics of studies

Study author (year) QA rating	Intervention domain	Country	Study design	Population	Sample size	Loss to follow-up	Length of observation
Cheng (2007) High	Health focused	Hong Kong	Randomized trial	MSK/pain	i1 = 46 c1 = 48	Not provided	4 weeks
Linton (1992) Moderate	Health focused	Sweden	Randomized trial	MSK/pain	i1 = 36 c1 = 30	Not provided	6 months (all subjects) 18 months (i1)
Norrefalk (2005) Moderate	Health focused	Sweden	Non-randomized trial	MSK/pain	i1 = 72 c1 = 14	i1 = 5 c1 = Not provided	1 year
Lidstrom (1992) High	Health focused	Sweden	Randomized trial	MSK/pain	i1 = 51 c1 = 52	Not provided	2 years
Hlobil 2005 High	Health focused	The Netherlands	Randomized trial	MSK/pain	i1 = 67 c1 = 67	i1 = 0 c1 = 0	1 year (RTW) 3 years (costs)
Verbeek (2002) High	Health focused	The Netherlands	Randomized trial	MSK/pain	i1 = 61 c1 = 59	Not provided	1 year
Whitfill (2010) High	Health focused	USA	Randomized trial	MSK/Pain	i1 = 58 c1 = 44	Not provided	1 year
Haig (1990) Moderate	Service coordination	USA	Cohort with historical comparison	MSK/pain	i1 = 61 c1 = 52	Not provided	1 year
McCluskey (2006) Moderate	Service coordination	United Kingdom	Non-randomized trial	MSK/pain	i1 = 81 i2 = 223 c1 = 214	Not provided	1 year
Ryan (1995) Moderate	Service coordination	Australia	Cohort with concurrent comparison	MSK/pain	Not provided	Not provided	6 years
van Oostrom (2010) High	Service coordination	The Netherlands	Randomized trial	Mental health	i1 = 73 c1 = 72	i1 = 0 c1 = 2	1 year
Anema (2004) Moderate	Work modification	Denmark, Germany, Israel, the Netherlands, Sweden & USA	Cohort with concurrent comparison	MSK/pain	i1 = 206, i2 = 299, i3 = 270 c1 = 311, c2 = 244, c3 = 291	Not provided	2 years
Hanson (2001) Moderate	Work modification	USA	Cohort with concurrent comparison	MSK/pain	i1 = 14, i2 = 29 c1 = 14	Not provided	1 year
Viikari-Juntura (2012) High	Work modification	Finland	Randomized trial	MSK/pain	i1 = 32 c1 = 31	i1 = 1 c1 = 5	1 year
Shaw (2006) Moderate	Work modification	USA	Randomized trial	MSK/pain	i1 = 11 c1 = 12	Not provided	14 months
Bernacki (2003) Moderate	Multi-domain	USA	Cohort with historical comparison	MSK/pain	i1 = 17k to 28k per annum (1993 to 1999) c1 = 16k to 17k per annum (1989 to 1992)	Not provided	10 years
Beutel (2005) Moderate	Multi-domain	Germany	Randomized trial	Mental health	i1 = 179 c1 = 87	i1 = 83 c1 = 2	2 years
Davis (2004) Moderate	Multi-domain	Canada	Cohort with historical and concurrent comparison	MSK/pain	i1 = 90 c1(hist.) = 345 c2(con.) = 53	Not provided	6 months
Jensen (1998) High	Multi-domain	Sweden	Cohort with concurrent comparison	MSK/pain	i1 = 67 c1 = 29	i1 = 9 c1 = 4	18 months
Lambeek (2010) High	Multi-domain	The Netherlands	Randomized trial	MSK/pain	i1 = 66 c1 = 68	i1 = 3 c1 = 7	1 year
Larson (2011) Moderate	Multi-domain	USA	Cohort with historical comparison	MSK/pain	i1 = 661 c1 = 713	Not provided	8 weeks
Nordstrom-Bjorverud (1998) Moderate	Multi-domain	Sweden	Cohort with historical comparison	MSK/pain	i1 = 34 c1 = 72	i1 = 0 c1 = 15	2–4 years (median: 2.8 years)
Yassi (1995) Moderate	Multi-domain	Canada	Non-randomized trial	MSK/pain	i1 = 60 c1 = 158	Not provided	2 years
Jensen (2013) High	Multi-domain	Denmark	Non-randomized trial	MSK/pain, mental health	i1 = 114 c1 = 86	i1 = 27 c1 = Not provided	2 years
Karlson (2010) Moderate	Multi-domain	Sweden	Non-randomized trial	Mental health	i1 = 74 c1 = 74	i1 = 0 c1 = 0	18 months
Anema (2007) Moderate	Health focused (i2), Work modification (i1), Multi-domain (i3)	The Netherlands	Randomized trial	MSK/pain	i1 = 96, i2 = 55, i3 = 27 c1 = 100, c2 = 57, c3 = 85	i1 = 10, i2 = 19, i3 = Not provided c1 = 0, c2 = 0, c3 = 0	1 year
Blonk (2006) Moderate	Health focused (i1), Multi-domain (i2)	The Netherlands	Randomized trial	Mental health	i1 = 40, i2 = 40 c1 = 42	i1 = 10, i2 = 10 c1 = 13	1 year
Hees (2013) High	Health focused (c1), Multi-domain (i1)	The Netherlands	Randomized trial	Mental health	i1 = 78 c1 = 39	i1 = 10 c1 = 6	18 months
Vlasveld (2013) High	Health focused (c1), Multi-domain (i1)	The Netherlands	Randomized trial	Mental health	i1 = 65 c1 = 61	i1 = 21* c1 = 31*	1 year
Arends (2013) High	Health focused (c1), Multi-domain (i1)	The Netherlands	Randomized trial	Mental health	i1 = 80 c1 = 78	i1 = 23* c1 = 28*	1 year
Kroger (2015) High	Health focused (c1), Multi-domain	Germany	Non-randomized trial	Mental health	i1 = 13 c1 = 13	i1 = 0 c1 = 0	1 year
Lagerveld (2012) High	Health focused (c1), Multi-domain (i1)	The Netherlands	Non-randomized trial	Mental health	i1 = 105 c1 = 103	i1 = 30 c1 = 23	1 year
Schene (2007) High	Health focused (c1), Multi-domain (i1)	The Netherlands	Randomized trial	Mental health	i1 = 32 c1 = 30	i1 = 8 c1 = 5	3.5 years
Karjalainen (2003) High	Health focused (i1), Multi-domain (i2)	Finland	Randomized trial	MSK/pain	i1 = 58 i2 = 55 c1 = 57	i1 = 0 i2 = 2 c1 = 3	2 years
Lemstra (2004) Moderate	Health focused (i2), Multi-domain (i1)	Canada	Cohort with historical and concurrent comparison	MSK/pain	i1 = 232, i2 = 232 c1 = 185, c2 = 285	Not provided	3 years
Loisel (1997) High	Health focused (i1), Work modification (i2), Multi-domain (i3)	Canada	Randomized trial with cross-over	MSK/pain	i1 = 31, i2 = 22, i3 = 25 c1 = 26	Not provided	6.4 years

*i* intervention group, *c* comparison group, *hist* historical, *con* concurrent

*Loss to follow-up only affected self-report measures. RTW data was available for all participants

#### Intervention Categorization

A diverse range of interventions were included. An intervention components inventory was created so medium to high quality studies could be aggregated into mutually exclusive categories; 12 unique intervention categories were developed (see Table 3) across four broad domains. Studies were allocated based on investigator consensus on the primary intervention objective. The four domains are:

**Table 3:** Level of evidence for workplace-based RTW interventions and accompanying messages

Levels of evidence (direction of effect)	Intervention (No. of H and M studies)	Outcome	Message
Strong (positive)	Multi-domain MSK interventions (4H, 10M)	Lost time	Implementing a multi-domain intervention (with components in at least 2 of the following domains: health-focused, service coordination, or work modification) can help reduce lost time for MSK and pain-related conditions
	Work-focused CBT for MH conditions (6H, 1M) Work-focused CBT for MH conditions (4H)	Lost time Cost	Implementing a work-focused CBT intervention can help reduce lost time and costs associated with work disability for mental health conditions
Strong (no effect)	CBT for MH conditions (6H, 1M)	Lost time	Implementing a traditional CBT intervention has no effect on reducing lost time for mental health conditions
Moderate (positive)	Graded activity (2H, 1M) Work accommodations (2H, 3M) Multi-domain MSK interventions (1H, 2M) Work-focused CBT for MH conditions (2H) Multi-domain MSK interventions (2H, 4M)	Lost time Lost time Work functioning Work functioning Cost	Consider implementing these interventions in practices if applicable to the work context
Limited (positive)	Work accommodations (1H, 1M) Health-focused multi-component (1H)	Cost Work functioning	Not enough evidence from the scientific literature to guide current policies/practices
Limited (no effect)	Work hardening (1H) Physician training (1H) RTW plan (1H, 1M) RTW plan (1H)	Work functioning Lost time Lost time Cost	Not enough evidence from the scientific literature to guide current policies/practices
Mixed	Work hardening (1H, 1M) Health-focused multi-component (3H, 2M) Graded activity (1H, 1M) Health-focused multi-component (2H)	Lost time Lost time Cost Cost	Not enough evidence from the scientific literature to guide current policies/practices
Insufficient	Case management (1M) Work accommodations (1M) Worker education/training (1M) Supervisor education/training (1M) Work hardening (1M)	Lost time Work functioning Cost Cost Cost	Not enough evidence from the scientific literature to guide current policies/practices

*H* high quality, *M* medium quality, *MSK* musculoskeletal or pain-related conditions, *CBT* cognitive behavioural therapy, *MH* mental health conditions, *RTW* return-to-work

1. Health-focused interventions. These interventions facilitate the delivery of health services to the injured worker either in the workplace or in settings linked to the workplace (e.g., visits to healthcare providers initiated by the employer/workplace). Specific health services intervention subcategories for which evidence synthesis was conducted include; graded activity/exercise, cognitive behavioural therapy, work hardening and multi-component health-focused interventions (which often included the above elements as well as: medical assessment, physical therapy, psychological therapy, occupational therapy).
2. Service coordination interventions. These interventions were designed to better coordinate the delivery of, and access to, services to assist RTW within and involving the workplace. Coordination involves attempts to improve communication within the workplace or between the workplace and the healthcare providers. Examples are development of RTW plans, case management and education and training.
3. Work modification interventions. These interventions alter the organization of work or introduce modified working conditions. Examples are: workplace accommodations such as provision of modified duties, modified working hours, supernumerary replacements, ergonomic adjustments or other worksite adjustments.
4. Multi-domain interventions. These interventions had multiple intervention components and included at least two of the three above intervention domains [e.g., interventions that involved graded activity in the workplace (health-focused domain) in addition to modified working conditions (work modification domain)].

Across the 36 studies, seven studies investigated health-focused interventions [24–32, 61–63], four studies examined service coordination interventions [33–35, 64–66], and four studies focused on work modification interventions [36–38, 67–69]. In addition, there were 21studies the review team felt were multi-domain interventions. The vast majority of these (n = 15) included components from all three domains [41, 42, 44–50, 54–60, 70–78, 80–85, 91, 92]. Two studies were focused on the health-focused and service coordination domains [43, 51–53], three studies included components from the health-focused and work modification domains [39, 40, 79, 87–90] and one study focused on intervention components from the service coordination and work modification domains [86]. Some multi-intervention studies (n = 5) compared interventions across more than one of these domains [56–60, 87–92].

#### RTW Outcome Categorization

Three RTW outcomes categories were derived from an inventory of outcome components:

1. Lost time: measures approximating the amount of time spent away from the workplace, or the rate of RTW amongst a group over a given time period. These include outcomes such as days from injury until first return to work, total duration of sick leave over a given time period, work status (working/not working) at a point in time, and recurrences of sick leave/work absence. These measures may be self-reported or collected from organisational or system records.
2. Work functioning: measures assessing the workers function in the workplace and health-related lost productivity. These include outcomes such as the self-rated work limitations questionnaire and estimates of productive working hours.
3. Costs: measures of work disability cost and time loss including costs of income replacement as well as the total cost of compensation paid (where such costs included income replacement costs).

There was one study with negative effects reported for both the lost time and disability costs outcomes [91, 92] in this review (Supplementary Table 3). The most common RTW outcome reported was lost time, which was included in 34 studies. There were 8 studies that examined work functioning outcomes and 15 studies that evaluated cost outcomes. Overall, positive effects were reported for at least one outcome in 29 of the 36 studies.

### Evidence Synthesis

Where appropriate, the interventions across the 36 studies were grouped into 12 different intervention categories within the four domains described above. Evidence synthesis for each category was determined and paired with practical messages (see Table 3 for a complete list of categories). The message content was determined through iterative stakeholder consultations to improve practicality. The messages were worded to help clarify the strength of the evidence, limit misinterpretation and increase user uptake.

Multi-domain interventions had a strong level of evidence showing a positive effect on the primary outcome of lost time associated with work disability. Fourteen studies [39–42, 44, 56–60, 70–89, 91, 92] targeted MSK or pain-related conditions. These four high and 10 medium quality studies presented a strong positive effect for comprehensive multi-domain interventions to reduce lost time (see Supplementary Table 3 for a more complete description of the intervention programs; see Table 3 for the evidence synthesis and practical messages for stakeholders). This strong level of evidence resulted in the following message for stakeholders: implementing a multi-domain intervention (i.e. with multiple health-focused, service coordination, and work modification components) can help reduce lost time for MSK and pain-related conditions.

In addition, seven multi-domain interventions for MH conditions [43, 45–55, 90] had a strong level of evidence. These six high and one medium quality studies offered cognitive behavioural therapy (CBT) focused on identifying work relevant solutions. Together, they presented a strong positive effect on reducing lost time for individuals with MH conditions. Four of these high quality studies [43, 47–53, 55] also found a strong positive effect for improving costs associated with work disability for these conditions (see Supplementary Table 3 and Tables 2, 3 for details). Together, these strong levels of evidence resulted in the following message: implementing a work-focused CBT intervention can help reduce lost time and costs associated with work disability for MH conditions.

One intervention category found a strong level of evidence of no effect on lost time for MH conditions. Seven studies (six high and one medium quality) [43, 45–55, 90] found that cognitive behavioural therapy alone offered no effect on lost time for MH conditions, leading to the following stakeholder message: implementing a traditional CBT intervention has no effect on reducing lost time for MH conditions (see Supplementary Table 3 and Tables 2, 3 for more details).

There was a moderate level of evidence for a positive effect on the primary outcomes for the following intervention domains: (see Supplementary Table 3, and Tables 2, 3 for details).

1. Health-focused interventions: graded activity programs (3 studies: 2 high and 1 moderate quality) [25–30, 87–89] were found to have a positive effect on reducing lost time.
2. Work modification interventions: work accommodations (5 studies: 2 high and 3 medium quality) [36–38, 58–60, 67, 68, 87–89] were found to have a positive effect on reducing lost time.
3. Multi-domain interventions for MSK or pain-related conditions were found to improve work functioning after RTW (3 studies: 1 high, 2 medium quality) [39, 40, 44, 70–75]; and were also shown to improve costs associated with work disability (2 high, 4 medium quality) [56–60, 70–75, 77, 80–85, 91, 92].
4. Multi-domain interventions for MH conditions (2 high quality studies) [45–50] were found to improve work functioning after RTW.

The key message for stakeholders arising from these moderate levels of evidence of a positive effect is: consider implementing these interventions if applicable to the work context.

The evidence for the primary outcomes across the remaining intervention categories (Health-focused multi-component (3H, 2M) [32, 56–63], work hardening (1H, 1M) [24, 91, 92], physician training (1H) [31], RTW plan (1H, 1M) [33–35, 64], case management (1M) [65], worker education/training (1M) [66], supervisor education/training (1M) [69]) resulted in limited, mixed or insufficient evidence as a result of either too few high quality studies available or from conflicting evidence across studies (Table 3). This resulted in the message: there is not enough evidence from the scientific literature to guide current policies or practices for several of these intervention categories. For a message to be provided for these interventions, more high quality consistent evidence is needed (Table 3).

## Discussion

The current review and evidence update gathers and synthesizes the scientific literature and presents practical messages for workplace parties and occupational health and safety practitioners. The review team consulted with these stakeholders to help ensure the messages were useful and applicable in practice.

The review identified 36 medium and high quality intervention studies that examined workplace-based RTW and disability management/support initiatives. The primary finding is strong evidence that multiple domain interventions are effective in improving RTW outcomes in workers with MSK, pain-related or MH conditions. In contrast, most single domain focused interventions have mixed or limited evidence to support their effectiveness. This result is aligned with one of the dominant theoretical paradigms in the work disability and return to work literature, the Sherbrooke model [93]. This model proposes that multi-disciplinary and multi-factorial interventions that seek to address an array of individual and societal factors that influence RTW are likely to be effective.

Combining newer studies with those from the original review [7] resulted in stronger evidence levels across a greater number of intervention categories. In addition, we were able to synthesize new evidence on intervention strategies to manage MH conditions in the workplace, which has emerged as an important area of concern for employers since the original review was published.

Our review identified that in most cases interventions were multi-faceted and included multiple intervention components, often operating across multiple domains (health focus, service coordination and work modification). This approach is different to the previous review [7], which sought to evaluate the effectiveness of discrete intervention components; leading to a different interpretation of the literature.

For example, the original review, Franche et al. [7], found a strong level of evidence for a positive effect of work accommodations, while in the current update only a moderate level of evidence was found. Of note, one of the interventions included in the original review examining work accommodation offers was reclassified in this review as a multi-domain intervention (of which work accommodations was only one of many components investigated) [80–85]. Among the five studies in this review looking at the effect of work accommodation on its own, two were rated as high quality [36–38, 58–60] and three were rated as medium quality [67, 68, 87–89]. According to our evidence synthesis algorithm (shown in Table 1), a minimum of three high quality studies was necessary to assign a strong level of evidence, which contributed to the change in level of evidence.

Although the types of interventions evaluated were diverse across the 36 studies, they could be grouped into one of four major domains, and 12 intervention categories, based on a consensus view of the primary intervention objective (i.e. health-focused, service coordination, work accommodation or multi-domain). Nearly 60% of these studies (n = 21) included multi-domain interventions, indicating that they included at least two of the three intervention domains mentioned above. Ninety-four percent of the included studies (n = 34) used an estimate of lost time from work as their primary RTW outcome variable. This is consistent with the broader RTW research literature in which lost time is often the outcome used to assess return to work status, despite the inherent limitations of this approach [94]. Other outcomes included work functioning and costs of work disability, but these were less commonly reported.

Our findings are consistent with other reviews that included workplace-based interventions [7, 95–97]; although reviews that focused on RCTs only and conducted meta-analyses found only moderate levels of evidence for workplace interventions [95–97]. While the current findings are consistent, our synthesis of workplace-based interventions for RTW in workers experiencing lost time from work due to MSK, pain-related and MH conditions includes practical messages for, and developed with, practitioners [17, 23].

This review highlighted a number of features of the RTW literature, and of workplace-based intervention studies in particular, worthy of comment. Fourteen of the 18 high quality studies were randomized trials, while only five of the 18 moderate quality studies were randomized trials. The majority of moderate quality studies were cohort studies with comparison groups. Due largely to their design, these studies were unable to ensure the presence of important quality standards such as blinding of participants and personnel, and allocation concealment. These moderate quality studies also suffered from quality limitations in that they were subject to attrition bias (uneven attrition and substantial loss to follow-up) and did not routinely assess compliance with the intervention. The review identified a group of 19 published randomized trials, which demonstrates that it is feasible to conduct such trials in the field. We also identified three non-randomized trials and one cohort study that were rated as high quality, and five randomized trials that were rated as moderate quality. Moving forward, a strong focus on study quality in addition to trial design is warranted.

It is now accepted that the system of compensating work-related injury can exert powerful influences on injured worker RTW [98]. Despite this, a recent systematic review identified that only a small proportion of studies including persons with compensable injury report on aspects of the compensation process [99]. The authors proposed that research involving persons with compensable conditions should include a description of system level factors such as compensation system structure and administration (e.g., source of funding); scheme eligibility (e.g., workforce coverage, claim coverage, waiting periods); scheme benefits and entitlements (e.g., level and duration of wage-replacement benefits); and case management (e.g., work capacity review, role of physician). Descriptions of system factors were often absent in the studies included in the present review, despite the study samples being predominantly workers with compensable injuries.

Due to the substantial heterogeneity across studies regarding intervention components, workplace contexts and study designs, a meta-analysis was not conducted. Instead, a best evidence synthesis (BES) approach [22] consistent with the original review [7] was used. While this approach has been criticized for being at risk of producing biased results [100], it is a transparent approach with clearly defined criteria to determine the level of evidence. This provides practitioners with useful information in addition to accessing the messages from the synthesis of studies. Practitioners can also more readily identify and consider relevant evidence from individual studies using this approach. This is especially practical when there are few studies available for a given intervention, as practitioners still need to act even when there is limited scientific evidence available to help guide their practice.

A particular strength of this review is the unique process of stakeholder engagement adopted throughout the review process. Our stakeholders provided guidance to ensure the review question was relevant, the search terms were comprehensive and the targeted literature identified was up-to-date. But more importantly, stakeholders helped us examine the findings from this review to determine the best wording for our key messages to facilitate uptake and dissemination of these evidence-based approaches for OHS practitioners and other workplace parties.

## Conclusions

Our synthesis update of the scientific literature identified 12 different types of interventions from 36 studies examining three broad RTW outcomes (i.e. lost time, work functioning and costs associated with work disability). There were several intervention types that did not meet the criteria for high or moderate levels of evidence across these different outcomes. However, we note that this does not mean that these interventions are not effective, only that there is insufficient evidence to support recommending these interventions to address RTW outcomes based on the scientific evidence.

Graded activity programs and work accommodations had a moderate level of evidence for a positive effect in reducing lost time associated with work disability. Practitioners should *consider* implementing graded activity programs and work accommodations in practices if applicable to the work context.

Cognitive behavioural therapy (CBT) programs focused on work relevant solutions for MH conditions had a strong level of evidence for a positive effect on both reducing lost time and costs associated with work disability. Additionally, there was a moderate level of evidence that these work-focused CBT programs had a positive effect on work functioning after RTW. We *recommend* implementing work-focused CBT interventions to help reduce lost time and costs associated with work disability for MH conditions. Practitioners should also *consider* implementing these programs to help improve work functioning after RTW for individuals with MH conditions.

Alternatively, there was a strong level of evidence indicating that traditional cognitive behavioural therapy programs for MH conditions have no effect on reducing lost time from work. We *recommend* practitioners should seek alternative interventions (such as work-focused CBT programs) to improve RTW after illness for MH conditions.

There was a strong level of evidence to support multi-domain interventions that include multiple components aimed at service coordination, work modification and improving worker health for reducing lost time associated with musculoskeletal injuries and pain-related conditions. Additionally, there was a moderate level of evidence that these multi-domain interventions had a positive effect on improving work functioning after RTW and reducing costs associated with work disability. We *recommend* implementing a multi-domain intervention (i.e. with health-focused, service coordination, and work modification components) to help reduce lost time for MSK and pain-related conditions. Practitioners should also *consider* implementing these programs to help improve work functioning and reduce costs associated with work disability for people with MSK or pain-related conditions.

## Electronic supplementary material

Below is the link to the electronic supplementary material.

Supplementary material 1 (DOCX 23 KB)

## References

[1] Waddell G, Burton AK (2006). Is work good for your health and well-being?.

[2] Black CM (2008). Working for a healthier tomorrow: Dame Carol Black’s review of the health of Britain’s working age population: presented to the Secretary of state for health and the Secretary of state for work and Pensions.

[3] Social Research Centre (2014). National return to work survey. 2013/14 summary research report (Australia and New Zealand).

[4] Associations of Workers’ Compensation Boards of Canada. AWCBC Key Statistical Measures Report. 2014. http://awcbc.org/. Accessed 13 Aug 2015.

[5] Berecki-Gisolf J, Clay FJ, Collie A, McClure RJ (2012). The impact of aging on work disability and return to work: insights from workers’ compensation claim records. J Occup Environ Med.

[6] Quinlan M, Mayhew C, Bohle P (2001). The global expansion of precarious employment, work disorganization, and consequences for occupational health: a review of recent research. Int J Health Serv.

[7] Franche RL, Cullen K, Clarke J, Irvin E, Sinclair S, Frank J (2005). Workplace-based return-to-work interventions: a systematic review of the quantitative literature. J Occup Rehabil.

[8] Loisel P, Buchbinder R, Hazard R, Keller R, Scheel I, Van Tulder M (2005). Prevention of work disability due to musculoskeletal disorders: the challenge of implementing evidence. J Occup Rehabil.

[9] MacEachen E, Clarke J, Franche RL, Irvin E (2006). Systematic review of the qualitative literature on return to work after injury. Scand J Work Environ Health.

[10] Pransky GS, Loisel P, Anema JR (2011). Work disability prevention research: current and future prospects. J Occup Rehabil.

[11] Furlan AD, Gnam WH, Carnide N, Irvin E, Amick BC, DeRango K (2012). Systematic review of intervention practices for depression in the workplace. J Occup Rehabil.

[12] Higgins JP, Green S. Cochrane handbook for systematic reviews of interventions. Version 5.1.0 (updated March 2011) ed. The Cochrane Collaboration; 2011.

[13] Adler DA, McLaughlin TJ, Rogers WH, Chang H, Lapitsky L, Lerner D (2006). Job performance deficits due to depression. Am J Psychiatry.

[14] Kessler RC, Barber C, Birnbaum HG, Frank RG, Greenberg PE, Rose RM (1999). Depression in the workplace: effects on short-term disability. Health Aff.

[15] Lerner D, Adler DA, Chang H, Lapitsky L, Hood MY, Perissinotto C (2004). Unemployment, job retention, and productivity loss among employees with depression. Psychiatr Serv.

[16] Greenberg PE, Stiglin LE, Finkelstein SN, Berndt ER (1993). The economic burden of depression in 1990. J Clin Psychiatry.

[17] Keown K, Van Eerd D, Irvin E (2008). Stakeholder engagement opportunities in systematic reviews: knowledge transfer for policy and practice. J Contin Educ Health Prof.

[18] Irvin E, Van Eerd D, Amick BC, Brewer S (2010). Introduction to special section: systematic reviews for prevention and management of musculoskeletal disorders. J Occup Rehabil.

[19] Evidence Partners (Internet). DistillerSR (electronic online systematic review software). Evidence Partners, Ottawa. 2015. http://distillercer.com/products/distillersr-systematic-review-software/. Accessed on Jan 2015.

[20] Kennedy CA, Amick BC, Dennerlein JT, Brewer S, Catli S, Williams R (2010). Systematic review of the role of occupational health and safety interventions in the prevention of upper extremity musculoskeletal symptoms, signs, disorders, injuries, claims and lost time. J Occup Rehabil.

[21] Shadish WR, Cook TD, Campbell DT (2002). Experimental and quasi-experimental designs for generalized causal inference.

[22] Slavin RE (1986). Best-evidence synthesis: an alternative to meta-analytic and traditional reviews. Educ Res.

[23] Institute for Work & Health (2007). Evidence and quality…Saying what works clearly!.

[24] Cheng ASK, Hung LK (2007). Randomized controlled trial of workplace-based rehabilitation for work-related rotator cuff disorder. J Occup Rehabil.

[25] Lindstrom I, Ohlund C, Eek C, Wallin L, Peterson L, Fordyce WE (1992). The effect of graded activity on patients with subacute low back pain: a randomized prospective clinical study with an operant-conditioning behavioral approach... including commentary by Nelson RM with author response. Phys Ther.

[26] Ohlund C, Lindstrom I, Eek C, Areskoug B, Nachemson A (1996). The causality field (extrinsic and intrinsic factors) in industrial subacute low back pain patients. Scand J Med Sci Sports.

[27] Staal JB, Hlobil H, Twisk JW, Smid T, Koke AJ, van Mechelen W (2004). Graded activity for low back pain in occupational health care: a randomized, controlled trial. Ann Intern Med.

[28] Hlobil H, Staal JB, Twisk J, Koke A, Ariens G, Smid T (2005). The effects of a graded activity intervention for low back pain in occupational health on sick leave, functional status and pain: 12-month results of a randomized controlled trial. J Occup Rehabil.

[29] Hlobil H, Uegaki K, Staal JB, De Bruyne MC, Smid T, van Mechelen W (2007). Substantial sick-leave costs savings due to a graded activity intervention for workers with non-specific sub-acute low back pain. Eur Spine J.

[30] Staal JB, Hlobil H, Koke AJ, Twisk JW, Smid T, van Mechelen W (2008). Graded activity for workers with low back pain: who benefits most and how does it work?. Arthritis Rheum.

[31] Verbeek JH WE, van Dijk FJ (2002). Early occupational health management of patients with back pain: a randomized controlled trial... including commentary by Vollinn E. Spine.

[32] Whitfill T, Haggard R, Bierner SM, Pransky G, Hassett RG, Gatchel RJ (2010). Early intervention options for acute low back pain patients: a randomized clinical trial with one-year follow-up outcomes. J Occup Rehabil.

[33] van Oostrom SH, van Mechelen W, Terluin B, de Vet HC, Anema JR (2009). A participatory workplace intervention for employees with distress and lost time: a feasibility evaluation within a randomized controlled trial. J Occup Rehabil.

[34] van Oostrom SH, van Mechelen W, Terluin B, de Vet HC, Knol DL, Anema JR (2010). A workplace intervention for sick-listed employees with distress: results of a randomised controlled trial. Occup Environ Med.

[35] van Oostrom SH, Heymans MW, de Vet HC, van Tulder MW, van Mechelen W, Anema JR (2010). Economic evaluation of a workplace intervention for sick-listed employees with distress. Occup Environ Med.

[36] Martimo KP, Kaila-Kangas L, Kausto J, Takala EP, Ketola R, Riihimaki H (2008). Effectiveness of early part-time sick leave in musculoskeletal disorders. BMC Musculoskelet Disord.

[37] Kausto J, Miranda H, Martimo KP, Viikari-Juntura E (2008). Partial sick leave: review of its use, effects and feasibility in the Nordic countries. Scand J Work Environ Health.

[38] Viikari-Juntura E, Kausto J, Shiri R, Kaila-Kangas L, Takala EP, Karppinen J (2012). Return to work after early part-time sick leave due to musculoskeletal disorders: a randomized controlled trial. Scand J Work Environ Health.

[39] Jensen IB, Bodin L (1998). Multimodal cognitive-behavioural treatment for workers with chronic spinal pain: a matched cohort study with an 18-month follow-up. Pain.

[40] Jensen IB, Nygren A, Lundin A (1994). Cognitive-behavioural treatment for workers with chronic spinal pain: a matched and controlled cohort study in Sweden. Occup Environ Med.

[41] Lambeek LC, van Mechelen W, Buijs PC, Loisel P, Anema JR (2009). An integrated care program to prevent work disability due to chronic low back pain: a process evaluation within a randomized controlled trial. BMC Musculoskelet Disord.

[42] Lambeek LC, van Mechelen W, Knol DL, Loisel P, Anema JR (2010). Randomised controlled trial of integrated care to reduce disability from chronic low back pain in working and private life. BMJ.

[43] Schene AH, Koeter MW, Kikkert MJ, Swinkels JA, McCrone P (2007). Adjuvant occupational therapy for work-related major depression works: randomized trial including economic evaluation. Psychol Med.

[44] Jensen AG (2013). A two-year follow-up on a program theory of return to work intervention. Work.

[45] Hees HL, Koeter MW, de VG, Ooteman W, Schene AH (2010). Effectiveness of adjuvant occupational therapy in employees with depression: design of a randomized controlled trial. BMC Public Health.

[46] Hees HL, de Vries G, Koeter MWJ, Schene AH (2013). Adjuvant occupational therapy improves long-term depression recovery and return-to-work in good health in sick-listed employees with major depression: results of a randomised controlled trial. Occup Environ Med.

[47] Vlasveld MC, Anema JR, Beekman AT, van MW, Hoedeman R, van Marwijk HW (2008). Multidisciplinary collaborative care for depressive disorder in the occupational health setting: design of a randomised controlled trial and cost-effectiveness study. BMC Health Serv Res.

[48] Vlasveld MC, Feltz-Cornelis CM, Ader HJ, Anema JR, Hoedeman R, van MW (2012). Collaborative care for major depressive disorder in an occupational healthcare setting. Br J Psychiatry.

[49] Vlasveld MC, Feltz-Cornelis CM, Ader HJ, Anema JR, Hoedeman R, van MW (2013). Collaborative care for sick-listed workers with major depressive disorder: a randomised controlled trial from the netherlands depression initiative aimed at return to work and depressive symptoms. Occup Environ Med.

[50] Goorden M, Vlasveld M, Anema J, Mechelen W, Beekman A, Hoedeman R (2014). Cost-utility analysis of a collaborative care intervention for major depressive disorder in an occupational healthcare setting. J Occup Rehabil.

[51] Arends I, van der Klink JJ, Bultmann U (2010). Prevention of recurrent sickness absence among employees with common mental disorders: design of a cluster-randomised controlled trial with cost-benefit and effectiveness evaluation. BMC Public Health.

[52] Arends I, Bultmann U, van RW, Groen H, van der Klink JJL (2013). Economic evaluation of a problem solving intervention to prevent recurrent sickness absence in workers with common mental disorders. PLoS One.

[53] Arends I, van der Klink JJL, van Rhenen W, de Boer MR, Bultmann U (2014). Prevention of recurrent sickness absence in workers with common mental disorders: results of a cluster-randomised controlled trial. Occup Environ Med.

[54] Kroger C, Bode K, Wunsch EM, Kliem S, Grocholewski A, Finger F (2015). Work-related treatment for major depressive disorder and incapacity to work: preliminary findings of a controlled, matched study. J Occup Health Psychol.

[55] Lagerveld SE, Blonk RW, Brenninkmeijer V, Wijngaards-de ML, Schaufeli WB (2012). Work-focused treatment of common mental disorders and return to work: a comparative outcome study. J Occup Health Psychol.

[56] Karjalainen K, Malmivaara A, Pohjolainen T, Hurri H, Mutanen P, Rissanen P (2003). Mini-intervention for subacute low back pain: a randomized controlled trial... including commentary by Pransky G. Spine.

[57] Karjalainen K, Malmivaara A, Mutanen P, Roine R, Hurri H, Pohjolainen T (2004). Mini-intervention for subacute low back pain: two-year follow-up and modifiers of effectiveness. Spine.

[58] Loisel P, Durand P, Abenhaim L, Gosselin L, Simard R, Turcotte J (1994). Management of occupational back pain: the Sherbrooke model. Results of a pilot and feasibility study. Occup Environ Med.

[59] Loisel P, Abenhaim L, Durand P, Esdaile JM, Suissa S, Gosselin L (1997). A population-based, randomized clinical trial on back pain management. Spine.

[60] Loisel P, Lemaire J, Poitras S, Durand M, Champagne F, Stock S (2002). Cost-benefit and cost-effectiveness analysis of a disability prevention model for back pain management: a six year follow up study. Occup Environ Med.

[61] Linton SJ, Bradley LA, Jensen I, Spangfort E, Sundell L (1989). The secondary prevention of low back pain: a controlled study with follow-up. Pain.

[62] Linton SJ, Bradley LA (1992). An 18-month follow-up of a secondary prevention program for back pain: help and hindrance factors related to outcome maintenance. Clin J Pain.

[63] Norrefalk JR, Svensson O, Ekholm J, Borg K (2005). Can the back-to-work rate of patients with long-term non-malignant pain be predicted?. Int J Rehabil Res.

[64] Haig AJ, Linton P, McIntosh M, Moneta L, Mead PB (1990). Aggressive early medical management by a specialist in physical medicine and rehabilitation: effect on lost time due to injuries in hospital employees. J Occup Med.

[65] McCluskey S, Burton AK, Main CJ (2006). The implementation of occupational health guidelines principles for reducing sickness absence due to musculoskeletal disorders. Occup Med.

[66] Ryan WE, Krishna MK, Swanson CE (1995). A prospective study evaluating early rehabilitation in preventing back pain chronicity in mine workers. Spine.

[67] Anema JR, Cuelenaere B, van der Beek AJ, Knol DL, de Vet HC, van Mechelen W (2004). The effectiveness of ergonomic interventions on return-to-work after low back pain; a prospective two year cohort study in six countries on low back pain patients sicklisted for 3–4 months. Occup Environ Med.

[68] Hanson CS, Shechtman O, Gardner-Smith P (2001). Ergonomics in a hospital and a university setting: the effect of worksite analysis on upper extremity work-related musculoskeletal disorders. Work.

[69] Shaw WS, Robertson MM, McLellan RK, Verma S, Pransky G (2006). A controlled case study of supervisor training to optimize response to injury in the food processing industry. Work.

[70] Bernacki EJ, Tsai SP (1996). Managed care for workers’ compensation: three years of experience in an “employee choice” state. J Occup Environ Med.

[71] Bernacki EJ, Guidera JA (1998). The effect of managed care on surgical rates among individuals filing for workers’ compensation. J Occup Environ Med.

[72] Green-McKenzie J, Parkerson J, Bernacki E (1998). Comparison of workers’ compensation costs for two cohorts of injured workers before and after the introduction of managed care. J Occup Environ Med.

[73] Bernacki EJ, Guidera JA, Schaefer JA, Lavin RA, Tsai SP (1999). An ergonomics program designed to reduce the incidence of upper extremity work related musculoskeletal disorders. J Occup Environ Med.

[74] Bernacki EJ, Guidera JA, Schaefer JA, Tsai S (2000). A facilitated early return to work program at a large urban medical center. J Occup Environ Med.

[75] Bernacki EJ, Tsai SP (2003). Ten years’ experience using an integrated workers’ compensation management system to control workers’ compensation costs. J Occup Environ Med.

[76] Beutel ME, Zwerenz R, Bleichner F, Vorndran A, Gustson D, Knickenberg RJ (2005). Vocational training integrated into inpatient psychosomatic rehabilitation-short and long-term results from a controlled study. Disabil Rehabil.

[77] Davis PM, Badii M, Yassi A (2004). Preventing disability from occupational musculoskeletal injuries in an urban, acute and tertiary care hospital: results from a prevention and early active return-to-work safely program. J Occup Environ Med.

[78] Larson MC, Renier CM, Konowalchuk BK (2011). Reducing lost workdays after work-related injuries: the utilization of athletic trainers in a health system transitional work program. J Occup Environ Med.

[79] Nordstrom-Bjorverud G, Moritz U (1998). Interdisciplinary rehabilitation of hospital employees with musculoskeletal disorders. Scand J Rehabil Med.

[80] Yassi A, Tate R, Cooper JE, Snow C, Vallentyne S, Khokhar JB (1995). Early intervention for back-injured nurses at a large Canadian tertiary care hospital: an evaluation of the effectiveness and cost benefits of a two-year pilot project. Occup Med (Lond).

[81] Yassi A, Khokhar J, Tate R, Cooper J, Snow C, Vallentyne S (1995). The epidemiology of back injuries in nurses at a large Canadian tertiary care hospital: implications for prevention. Occup Med.

[82] Cooper JE, Tate R, Yassi A (1997). Work hardening in an early return to work program for nurses with back injury. Work.

[83] Cooper JE, Tate RB, Yassi A (1998). Components of initial and residual disability after back injury in nurses. Spine.

[84] Yassi A (1998). Utilizing data systems to develop and monitor occupational health programs in a large Canadian hospital. Methods Inf Med.

[85] Tate RB, Yassi A, Cooper J (1999). Predictors of time loss after back injury in nurses. Spine.

[86] Karlson B, Jonsson P, Palsson B, Abjornsson G, Malmberg B, Larsson B (2010). Return to work after a workplace-oriented intervention for patients on sick-leave for burnout–a prospective controlled study. BMC Public Health.

[87] Anema JR, Steenstra IA, Bongers PM, de Vet HC, Knol DL, Loisel P (2007). Multidisciplinary rehabilitation for subacute low back pain: graded activity or workplace intervention or both? A randomized controlled trial. Spine.

[88] Steenstra IA, Anema JR, Bongers PM, de Vet HC, van Mechelen W (2003). Cost effectiveness of a multi-stage return to work program for workers on sick leave due to low back pain, design of a population based controlled trial [ISRCTN60233560]. BMC Musculoskelet Disord.

[89] Steenstra IA, Anema JR, van Tulder MW, Bongers PM, De Vet HCW, van Mechelen W (2006). Economic evaluation of a multi-stage return to work program for workers on sick-leave due to low back pain. J Occup Rehabil.

[90] Blonk RWB, Brenninkmeijer V, Lagerveld SE, Houtman ILD (2006). Return to work: a comparison of two cognitive behavioural interventions in cases of work-related psychological complaints among the self-employed. Work Stress.

[91] Lemstra M, Olszynski WP (2003). The effectiveness of standard care, early intervention, and occupational management in worker’s compensation claims. Spine.

[92] Lemstra M, Olszynski WP (2004). The effectiveness of standard care, early intervention, and occupational management in Workers’ Compensation claims: part 2. Spine.

[93] Costa-Black KM, Feuerstein M, Loisel P (2013). Work disability models: past and present. Handbook of work disability.

[94] Vogel AP, Barker SJ, Young AE, Ruseckaite R, Collie A (2011). What is return to work? An investigation into the quantification of return to work. Int Arch Occup Environ Health.

[95] Nieuwenhuijsen K, Faber B, Verbeek JH, Neumeyer-Gromen A, Hees HL, Verhoeven AC (2014). Interventions to improve return to work in depressed people. Cochrane Database Syst Rev.

[96] Schandelmaier S, Ebrahim S, Burkhardt SC, de Boer WE, Zumbrunn T, Guyatt GH (2012). Return to work coordination programmes for work disability: a meta-analysis of randomised controlled trials. PloS one.

[97] van Vilsteren M, van Oostrom SH, de Vet HC, Franche RL, Boot CR, Anema JR. Workplace interventions to prevent work disability in workers on sick leave. Status and date: new search for studies and content updated (conclusions changed), The Cochrane Library. 2015. 10.1002/14651858.CD006955.pub3 PMC9297123 26436959

[98] Kilgour E, Kosny A, McKenzie D, Collie A (2014). Interactions between injured workers and insurers in workers’ compensation systems: a systematic review of qualitative research literature. J Occup Rehabil.

[99] Clay FJ, Berecki-Gisolf J, Collie A (2014). How well do we report on compensation systems in studies of return to work: a systematic review. J Occup Rehabil.

[100] Verbeek J, Husman K, Van Dijk F, Jauhiainen M, Pasternack I, Vainio H (2004). Building an evidence base for occupational health interventions. Scand J Work Environ Health.

