# Supplementary material for: Effectiveness of Workplace Interventions in Return-to-Work for Musculoskeletal, Pain-Related and Mental Health Conditions: An Update of the Evidence and Messages for Practitioners
Source: J Occup Rehabil. 2017 Feb 21;28(1):1–15. doi: 10.1007/s10926-016-9690-x (PMC5820404; doi:10.1007/s10926-016-9690-x)
Supplement: Supplementary file 1 — Supplementary material 1 (DOCX 23 KB) [file 10926_2016_9690_MOESM1_ESM.docx]

**List of Supplementary Tables for online publication**

1. Supplementary Table 1: List of search terms included in this review.

2. Supplementary Table 2: Methodological quality of studies included in this review.

3. Supplementary Table 3: Classification of intervention subcategories for studies included in this review.

Supplementary Table 1: List of search terms included in this review.

| **Search term area** | **List of terms** |
| --- | --- |
| Population (workers) | claimant(s), client(s), employee(s), employment, labo(u)rer(s), occupation(s), occupational, work site, work* based, worker(s), workforce, workplace |
| Population (injury / condition) | absentee(s), occupational diseases/rehabilitation, occupational diseases/therapy, occupational illness(es), presentee(s), work disability, work injuries, work injury, worker illness(es), workers compensation, workers compensation/workplace injury, workplace injuries |
| Intervention | absence management, alternative work, attendance management, care protocols, case conference, case management, case management plan, claims management plan, claims review, clinical practice guidelines, co-worker, collaboration, communication, communication with employer(s), communication with health care, communication with healthcare, communication with work place(s), communication with workplace(s), disability management, disability prevention, disclosure management, early contact, employee assistance, employer accommodation, employer contact, ergonomic intervention, flexible work(er), functional abilities evaluation, functional ability evaluation, functional capacity assessment, functional capacity evaluation, graded activity, graded work, graduated hours, health care provider training, healthcare provider training, human resource training, injury management, intervention  job accommodation, labo(u)r market re-entry, labo(u)r market re-entry, light duties, light duty, light work, LMR, long term disability, long term insurance plan, modified duties, modified duty, modified work, motivational interviewing, occupational rehabilitation, re-employ(ment), reasonable accommodation, reasonable adjustment  reemploy(ment), return employees to work, return to work, second employer program, self-management training, short term disability,  suitable duties, Supervisor, supervisor training, supportive co-workers, supportive colleague(s), supportive coworkers, supportive manager, supportive supervisor, vocational assessment, vocational rehabilitation, wage replacement, work accommodation, work adjustment, work conditioning, work hardening, work program(s), work programme(s), work re-integration, work reintegration, work role functioning, work visit, work* accommodation, work* intervention*, work* trial(s), work* re-integration, work* reintegration, workplace linked, worksite visit |
| Outcome | Absenteeism, alternative work, attendance (at work), benefit duration, communication with employer(s), communication with health care, communication with healthcare, communication with work place(s), communication with workplace(s), compensation claims cost(s), compensation costs, continuance costs, continuance rate, fit note, functional limitation(s), graded activity, Health, health-related work role functioning, health status, injury experience, labo(u)r market re-entry, labo(u)r market re-entry, liability reduction, LMR, long term disability, long term insurance plan, lost time, lost workday(s), maintenance at work, physical capacity, precarious work arrangement, Presenteeism, re-employ(ment), re-injury, Reinjury, reasonable accommodation, Recurrence, redeployed with job, reemploy(ment), Relapse, return employees to work, return to work, second employer program, secondary injury, short term disability, sick leave, sick listed, sickness absence, sickness related absence, social exclusion, suitable duties, suitable employment, supportive co-workers, supportive colleague(s), supportive coworkers, supportive manager, supportive supervisor, sustainable employment, time loss, time lost, time on benefit, wage replacement, work accommodation, work ability, workability, work capacity, work disability, work limitation(s), work maintenance, work participation, work re-integration, work readiness, work reintegration, work role functioning, worker(s) re-integration, worklessness. |

Supplementary Table 2: Methodological quality criteria applied to studies in this review.

| **Systematic review methodological quality criteria (question weight)** |
| --- |
| Q1. Is the research question/objective clearly stated? (2)  Q2. Was a primary hypothesis clearly stated? (1)  Q3. Were comparison group(s) used? (3)  Q4. Was an intervention allocation randomized? (3)  Q5. Was allocation concealment adequate? (1)  Q6. Were sample inclusion/exclusion criteria adequately described? (2)  Q7. Was recruitment (or participation) rate reported and adequate? (2)  Q8. Was the sampling frame representative of the target population? (1)  Q9. Did the author(s) examine whether important differences existed between those who participated and those who did not? (1)  Q10. Were pre-intervention (baseline) characteristics described and equivalent between groups? (2)  Q11. Was loss to follow up (attrition) significant and problematic? (2)  Q12. Did attrition affect groups equally? (2)  Q13. Did the author(s) compare post intervention those who dropped out to those who remained in the study? (2)  Q14. Was the intervention process adequately described to allow for replication? (2)  Q15. Was there any potential for contamination? (2)  Q16. Was there any potential for co-intervention? (2)  Q17. Were the effects of the intervention on key intervention mechanisms described? (1)  Q18. Was compliance with the intervention in all groups described and adequate? (1)  Q19. Was blinding of participants and personnel adequate? (1)  Q20. Were the instruments used to assess the outcomes valid and reliable? (3)  Q21. Were all important outcomes described at baseline and follow-up? (2)  Q22. Was the proportion of missing outcome data greater than 40% or substantially different between groups? (2)  Q23. Was there adjustment for important covariates, confounders or pre-intervention differences (if necessary)? (2)  Q24. Were the statistical analyses optimized for best results? (1)  Q25. Were all participants’ outcomes analyzed by the groups to which they were originally allocated (intention-to-treat analysis)? (2)  Q26. Was there a direct between group comparison? (3) |

Supplementary Table 3: Classification of intervention subcategories for studies included in this review.

| **Study Author (year)  QA rating** | **Intervention category** | **Intervention Components** | **Intervention Effects** |
| --- | --- | --- | --- |
| Cheng (2007) High | Health Focused | Work hardening | Lost time: Positive effect Work fx: No effect |
| Linton (1992) Moderate | Health Focused | Multi-component health-focused with:  Physical therapy, Occupational therapy, Psychological therapy | Lost time: No effect |
| Norrefalk (2005) Moderate | Health Focused | Multi-component health-focused with:  Medical assessment, Physical therapy, Occupational therapy, Psychological therapy | Lost time: No effect |
| Lindstrom (1992) High | Health Focused | Graded activity/exercise | Lost time: Positive effect |
| Hlobil (2005) High | Health Focused | Graded activity/exercise | Lost time: Positive effect Costs: Positive effect |
| Verbeek (2002) High | Health Focused | Occupational physician training | Lost time: No effect |
| Whitfill (2010) High | Health Focused | Multi-component health-focused with:  Graded activity/exercise, Medical assessment, Physical therapy, Occupational therapy, Psychological therapy | Lost time: Positive effect Work fx: Positive effect |
| Haig (1990) Moderate | Service Coordination | RTW planning & coordination | Lost time: No effect |
| McCluskey (2006) Moderate | Service Coordination | Case-management | Lost time: Positive effect (i1),  No effect (i2) |
| Ryan (1995) Moderate | Service Coordination | Worker education/training | Costs: Positive effect |
| van Oostrom (2010) High | Service coordination | RTW planning & coordination | Lost time: No effect Costs: No effect |
| Anema (2004) Moderate | Work Modification | Work accommodation | Lost time: Positive effect |
| Hanson (2001) Moderate | Work Modification | Work accommodation | Lost time: No effect Work fx: No effect |
| Viikari-Juntura (2012) High | Work Modification | Work accommodation | Lost time: Positive effect |
| Shaw (2006) Moderate | Work Modification | Supervisor education/training | Costs: No effect |
| Bernacki (2003)  Moderate | Multi-domain | Multi-component health-focused with:  Medical Assessment, Physical Therapy, Occupational Therapy  RTW planning & coordination  Work accommodation | Lost time: Positive effect Work fx: Positive effect Cost: Positive effect |
| Beutel (2005) Moderate | Multi-domain | Multi-component health-focused with:  Graded activity/exercise, Medical assessment,  Psychological therapy, Physical therapy  RTW planning & coordination  Worker education/training  Work accommodation | Lost time: Positive effect |
| Davis (2004) Moderate | Multi-domain | Multi-component health-focused with: Graded activity/exercise, Medical assessment, Physical therapy, Occupational therapy  RTW planning & coordination  Case management Worker education/training  Work accommodation | Lost time: Positive Costs: No effect |
| Jensen (1998) High | Multi-domain | Multi-component health-focused with:  Graded activity/exercise, Physical therapy, Occupational therapy, Psychological therapy  Supervisor education/training | Lost time: No effect Work fx: Positive effect |
| Lambeek (2010) High | Multi-domain | Multi-component health-focused with:  Graded activity/exercise, Medical assessment, Physical therapy, Occupational therapy  Worker education/training  Work accommodation  Supervisor education/training | Lost time: Positive effect |
| Larson (2011) Moderate | Multi-domain | Multi-component health-focused with:  Graded activity/exercise, Medical assessment, Physical therapy  RTW planning & coordination  Work accommodation | Lost time: Positive effect |
| Nordstrom-Bjorverud (1998) Moderate | Multi-domain | Multi-component health-focused with:  Graded activity/exercise, Work hardening,  Medical assessment, Physical therapy,  Occupational therapy  Worker education/training  Supervisor education/training | Lost time: Positive effect |
| Yassi (1995)  Moderate | Multi-domain | Multi-component health-focused with:  Graded activity/exercise, Work hardening,  Medical assessment, Physical therapy Occupational therapy  RTW planning & coordination  Case management Work accommodation | Lost time: Positive effect  Cost: Positive effect |
| Jensen (2013) High | Multi-domain | Multi-component health-focused with:  Cognitive behavioural therapy, Graded activity/exercise, Physical therapy  Worker education/training  Supervisor education/training | Lost time: Positive effect Work fx: Positive effect |
| Karlson (2010) Moderate | Multi-domain | RTW planning & coordination  Worker education/training  Work accommodation  Supervisor education/training | Lost time: Positive effect |
| Anema (2007) Moderate | Health Focused (i2),  Work Modification (i1),  Multi-domain (i3) | Graded activity/exercise (i2, i3)  RTW planning & coordination (i1, i3)  Work accommodation (i1, i3) | Lost time: Positive effect (i1), Negative effect (i2), No effect (i3)  Cost: No effect (i1, i2, i3) |
|  |  |  |  |
| Blonk (2006) Moderate | Health Focused (i1), Multi-domain (i2) | Cognitive behavioural therapy (i1, i2)  Work accommodation (i2) | Lost time: Positive effect (i2), No effect (i1) |
| Karjalainen (2003) High | Health Focused (i1),  Multi-domain (i2) | Multi-component health-focused with:  Graded activity/exercise (i1, i2), Medical assessment (i1, i2), Physical therapy (i1, i2)  Worker education/training (i2)  Supervisor education/training (i2) | Lost time: Positive effect (i1), No effect (i2) Cost: No effect (i1, i2) |
| Hees (2013) High | Health Focused (c1)  Multi-domain (i1) | Multi-component health-focused with:  Cognitive behavioural therapy, Work hardening, Medical assessment, Physical therapy  RTW planning & coordination  Supervisor education/training | Lost time: No effect for overall RTW, Positive effect for RTW in good health Work fx: Positive effect |
| Vlasveld (2013) High | Health Focused (c1)  Multi-domain (i1) | Cognitive behavioural therapy  RTW planning & coordination  Work accommodation | Lost time: No effect  Cost: Positive effect |
| Arends (2013) High | Health Focused (c1)  Multi-domain (i1) | Cognitive behavioural therapy  RTW planning & coordination | Lost time: Positive effect Work fx: Positive effect Cost: No effect |
| Kroger (2015) High | Health Focused (c1)  Multi-domain (i1) | Cognitive behavioural therapy  RTW planning & coordination  Work accommodation | Lost time: Positive effect |
| Lagerveld (2012) High | Health Focused (c1)  Multi-domain (i1) | Cognitive behavioural therapy  RTW planning & coordination  Work accommodation | Lost time: Positive effect Cost: Positive effect |
| Schene (2007) High | Health Focused (c1)  Multi-domain (i1) | Multi-component health-focused with:  Cognitive behavioural therapy, Work hardening,  Physical therapy  RTW planning & coordination | Lost time: Positive effect  Psych fx: Positive effect Cost: Positive effect |
| Lemstra (2004) Moderate | Health Focused (i2),  Multi-domain (i1) | Multi-component health-focused with:  Graded activity/exercise (i1), Medical assessment (i2), Physical therapy (i2), Work hardening (i2)  RTW planning & coordination (i1)  Worker education/training (i1) Work accommodation (i1) | Lost time: Positive effect (i1), Negative effect (i2) Cost: Positive effect (i1) Negative effect (i2) |
| Loisel (1997) High | Health Focused (i1), Work Modification (i2), Multi-domain (i3) | Multi-component health-focused with:  Work hardening (i1, i3), Medical assessment (i1, i3), Physical therapy (i1, i3),  Psychological therapy (i1, i3)  RTW planning & coordination (i1, i3) Worker education/training (i1, i3) Work accommodation (i2, i3) | Lost time: Positive effect (i2. i3), No effect (i1) Cost: Positive effect (i1, i2, i3) |
